# The short-term effect on alliance and satisfaction of using patient feedback scales in mental health out-patient treatment. A randomised controlled trial

**DOI:** 10.1186/1472-6963-12-348

**Published:** 2012-10-03

**Authors:** Marit By Rise, Lasse Eriksen, Hilde Grimstad, Aslak Steinsbekk

**Affiliations:** 1 Department of Public Health and General Practice, Norwegian University of Science and Technology, Trondheim, Norway; 2 Nidaros Regional Psychiatric Centre, St. Olav’s University Hospital, Trondheim, Norway; 3 Department of Neuromedicine, Norwegian University of Science and Technology, Trondheim, Norway

**Keywords:** Patient participation, Mental health care, Outcome assessment (Health Care), Professional-patient relations

## Abstract

**Background:**

The main aim was to investigate the effect of using two brief feedback scales in mental health out-patient treatment six weeks after starting treatment, compared to treatment as usual. Hypotheses were that use of feedback scales would improve treatment alliance and patient satisfaction.

**Methods:**

An open parallel-group randomised controlled trial was conducted in an out-patient unit in a mental health hospital in Central Norway. Eight therapists trained in using the feedback scales in the Partners for Change Outcome Management System (PCOMS) treated the intervention group. Seventeen therapists treated the controls, providing treatment without using feedback scales. The main outcome measures were treatment alliance and patient satisfaction.

**Results:**

Seventy-five patients participated. There were no differences between the groups in the intention to treat (ITT) analyses on alliance (mean difference = 0.08, 95% CI −0.44, 0.59, p = 0.760) or satisfaction (mean difference = 0.24, 95% CI −1.85, 2.32, p = 0.819), and no statistically significant differences between the groups in the per protocol (PP, n = 58) analyses on alliance (mean difference = 0.32, 95% CI −0.84, 3.16, p = 0.137) or satisfaction (mean difference = 1.16, 95% CI −0.84, 3.16, p = 0.248) six weeks after the treatment started. The effect size in favour of the PCOMS group increased from 0.07 for alliance and 0.06 for satisfaction in the intention to treat analysis to 0.40 on alliance and 0.31 for satisfaction in the per protocol analysis. Among the other outcomes, the PCOMS group had better motivation for treatment (estimated mean difference ITT: 0.29, 95% CI 0.00 to 0.57, p = 0.05, PP: 0.28, 95% CI 0.04, 0.52, p = 0.024).

**Conclusion:**

Six weeks after starting treatment, there were no effects on alliance and satisfaction from using two brief feedback scales. Since the per protocol analyses showed higher effect sizes, future investigations in a larger study with longer follow-up are warranted.

## Background

User participation is highly valued and encouraged in the Western world, and there are several proposed benefits from user participation in health care [1-5]. User participation is commonly described on two levels: on a system level when users are representatives in boards and groups, and on an individual level when patients are being involved in discussions and decisions regarding their own treatment. Many have described and defined user participation [6-13]. These definitions include patients’ involvement in their own treatment [7,14], patients’ right to be involved in decision-making [9], patient’s implementation and management of their own care [12], and a change of patient role from passive recipients to active participants [15]. These definitions share one important aspect: the patient’s perspective and influence on his or her treatment. This is in line with one of the main arguments for user participation in general, that involving the patients’ view can improve the quality of the health services

Patients and professionals perceive the quality of health care differently, since patients focus more on access, responsiveness, good communication, and information, as well as appropriate treatment, relief of symptoms, and improved health [11]. To measure patient satisfaction through surveys is the most common way to assess patients’ view on health care services [11,16]. Patient satisfaction is also one of the most common outcome measures when interventions intended to enhance individual patient participation are investigated [17,18]. Although many have argued that patient satisfaction is insufficiently defined [11,16,19], satisfaction is widely assessed in several aspects of health care; in the health system as a whole, in specific areas of health care i.e. general practice, in specific health care organisations such as hospital units, for specific clinicians, and for specific treatment approaches [11].

Regardless of the health care setting, the relationship between the patient and the provider is one of the most important factors affecting patient satisfaction [16]. Improving interpersonal issues is therefore highly recommended to enhance patient satisfaction [16]. Caring and respectful relationships between patient and provider are vital for patient satisfaction [20]. The relationship between patient and provider has been strongly emphasised in mental health care, and has been described as encompassing three parts: a working alliance, a transference configuration, and a real relationship [21,22]. The working alliance is considered to be the most fundamental for effective treatment [21]. Although the term treatment or working alliance originated in psychoanalysis, it can be generalized to all forms of psychotherapy [23]. Treatment or working alliance is described as the bond of collaboration and affection between patient and professional [24], and as a concept includes three features: that the patient and provider agree on goals, that they assign tasks, and that they develop bonds [23]. Research has repeatedly shown that there is a consistent relationship between a strong treatment alliance and a good treatment outcome [24].

Many have argued for systematically assessing feedback from patients during treatment in mental health care [25-29]. Such feedback consists of how the patients perceive the treatment outcome and how the patients experience the treatment session. Systematically assessing feedback from patients is part of an increased focus on the patient’s individual progress during treatment [25]. The main question is thus not whether a treatment works in general, but whether this specific treatment works for this specific patient [25]. The main argument for assessing feedback is twofold. Firstly, when given knowledge of the patient’s situation at the beginning of treatment, it is possible to predict the expected course of change for a successful treatment process [25]. Monitoring outcomes during treatment makes it possible to detect any deviations from the expected course, and this is vital to ensure good treatment outcomes and to prevent drop-out [30]. Secondly, measurements of the patient’s perception of the outcome and the consultations in every treatment session can be used actively to determine whether the current treatment is appropriate, whether further treatment is needed, and to alert the therapist when the patient is not progressing as expected [25,29].

Although these arguments are directed at improving the outcome from treatment, the process of asking for and discussing patient feedback during treatment strongly involve the patient’s perspective and thereby increase the patient’s participation both in the consultations and in the treatment process as a whole. Others have previously described interventions where patients’ rating of health status and quality of life are fed back to the providers as enhancing patient participation [17]. Given that user participation means including the patient’s perspective [7,13,14], it would be reasonable for the patient to partake in evaluation and the management of the treatment process. Since participation in the decision-making process is emphasised as a vital part of user participation [7,10,13,31], participation would mean that the patient is involved in deciding whether the current treatment should continue or be altered. Exchanging information through a dialogue between patient and therapist has also been emphasised as a core aspect of user participation [13].

Increasing participation has also been described as a redistribution of power [32]. Inviting the patient to partake in evaluation and decision-making is a concrete way to involve the patient more, and would transfer some power over the treatment process from the professional to the patient. To assess and collect comprehensible feedback data, and to monitor these data during the treatment process, makes it feasible for the patient and provider to evaluate and discuss treatment progress as equal partners. Concretely assessing feedback systematically invites the patient to exchange information with the provider, to evaluate the treatment effect and the consultation, to discuss potential improvements, and to determine whether the treatment approach is appropriate or should be altered. Systematically assessing and monitoring feedback from patients on the treatment outcome is thus a practical tool of user participation on the individual level.

Effort has been made to simplify the process of collecting and using patient feedback data, and to make it practical for both patients and professionals. Feedback systems have been developed to monitor the treatment progress from the patients’ point of view, i.e. the Outcome Questionnaire-45 (OQ-45) [33] and the Partners for Change Outcome Management System (PCOMS) [30]. These systems use standardised feedback scales to collect the patient’s views on outcomes in every consultation, and the results are discussed by the patient and the therapist during the treatment process [29]. A meta-analysis of studies on the effect of the OQ-45 and the PCOMS showed that patients who experienced little progress during treatment were detected through systematically assessing feedback, and they obtained better treatment outcomes than patients did where feedback was not assessed [29].

While the OQ-45 includes a rather extensive scale with 45 items, the Partners for Change Outcome Management System (PCOMS) includes two short feedback scales with four items each: the Outcome Rating Scale (ORS) and the Session Rating Scale (SRS) [30]. The ORS is used at the beginning of each treatment session to assess the patients’ rating of treatment outcome last week, or since the last session. The SRS is used at the end of each session to assess the patients’ rating of the current session. Only a few randomised controlled trials have investigated the effect of using the brief feedback scales in the PCOMS [29]. These trials have investigated effects in mental health treatment [34], in couples therapy [35,36], and in training and supervision of psychology students [37]. All four trials focused on treatment outcome measured by the ORS and found that using the PCOMS scales during treatment was superior to treatment as usual.

### Aim and hypotheses

To discuss the patient’s perception of the treatment outcome in some manner is widely acknowledged as an important and inherent part of all treatment for mental health problems. Using concrete feedback scales where the patient is repeatedly asked to evaluate and rate the treatment and the consultations, is a more definite way to assess and collect the patient’s views. Scales are a quantified form of patient feedback which can be used in further discussions on how to improve the treatment. Collecting feedback scales could also be considered a strong and definite signal that the patient’s views matter. It would therefore be of interest whether routinely using feedback scales to assess and monitor the patient’s views increases the alliance and satisfaction in early phases of treatment. Treatment including the use of feedback scales should therefore be compared to usual treatment, where feedback scales are not used. No studies so far have investigated the short-term effect of using brief feedback scales, such as the PCOMS, on treatment alliance and patient satisfaction.

The aim was therefore to investigate the short-term effects on treatment alliance and patient satisfaction from using the PCOMS scales (ORS and SRS) in out-patient mental health treatment, compared to treatment without using feedback scales (treatment as usual).

One of the arguments for using feedback scales is that a strong treatment alliance increases the possibility for a good treatment outcome [26]. Treatment alliance is considered to be an important predictor for outcome in mental health care [30,38,39], and it is found that the degree of treatment alliance can be established after only a few consultations [38]. Hypothesis 1 was therefore that the use of the PCOMS scales (ORS and SRS) would lead to stronger treatment alliance than treatment as usual, six weeks after starting treatment. In this study we defined treatment alliance as the patient’s perception of a relational bond and good collaboration with the therapist [40].

It has also been argued that using the ORS and SRS scales to ask patients for feedback during treatment helps foster a cooperative and accountable relationship between patient and professional [26]. The relationship between patient and provider is the most influential factor on patient satisfaction [41,42], and it would be reasonable to believe that the use of feedback scales would lead to improved patient satisfaction. Hypothesis 2 was therefore that the use of the PCOMS scales (ORS and SRS) would lead to a higher degree of patient satisfaction than treatment as usual, six weeks after starting treatment.

Secondary outcomes were mental health symptoms, patient activation, health-related quality of life, patient motivation, and patient participation.

## Methods

This was an open, randomised parallel-group controlled trial performed according to the principles of the Helsinki Declaration. The Regional Committee for Medical and Health Research Ethics, Central Norway, and the hospital’s management approved of the study. Participants had to sign an informed consent and were informed that they could withdraw during the study. Data collection was conducted from February 2010 to March 2011.

The study was carried out in an out-patient unit in a mental health hospital in Central Norway (Trondheim). The hospital, a part of St. Olav’s Hospital Trust, covers a catchment area of 96,000 people, with urban and semi-rural areas including parts of a large Norwegian city. The out-patient unit treats patients with all types of mental health diagnoses that do not require hospitalisation.

### Participants

All patients offered treatment at the out-patient unit between six weeks and three months after referral were eligible. The lower limit of six weeks was needed to allow for baseline assessment, randomisation, and treatment allocation in the units. There were no exclusion criteria.

All therapists at the out-patient unit who provided and were responsible for individual treatment were eligible for participating in the study.

### Recruitment

Invitation to participate was mailed to the patients together with the treatment approval letter. One week after the invitation was mailed an employee at the department or the 1^st^ author (MBR) phoned the patients asking if they wanted to participate in the study. Those who accepted were invited to meet with the 1^st^ author. During this meeting, oral and written information was given and a written consent was signed. Three patients wanted to participate without meeting with the researcher. They were informed by phone, received the written consent by mail, and returned it by mail.

All 29 therapists in the out-patient unit who provided and were responsible for individual treatment were invited to participate in the study. The invitation informed that they must be willing to receive training in use of the PCOMS scale on two specific days. Ten therapists volunteered to participate in the study, but only 8 could participate in the training at the given times. The two therapists who weren’t able to participate in the training sessions and six other therapists were recruited to participate in treating the treatment as usual group. The six therapists who had not volunteered were asked by the unit manager to participate. Due to administrative turn-over and other unanticipated difficulties in allocating control patients to therapists during the trial, the group of treatment as usual therapists had to be extended to 17 during the study. In total 86% of all therapists in the out-patient units at the hospital who provided individual treatment participated in the study.

### Intervention

The intervention therapists were trained to administer the feedback system Partners for Change Outcome Management System (PCOMS) [30] during the treatment they usually provide. PCOMS therapists received 12 h of training during two days, with four weeks apart, with respectively eight and four hours of training. In addition, the therapists could contact the instructor at their own will. The training the PCOMS therapists received was similar to previous studies [34-37], and in accordance with training given for clinical purposes to improve patient functioning and progress [43]. Training was given by an experienced instructor who did not participate in the research group. The instructor has been trained by the founders of the PCOMS, and has extensive experience both from using the system in clinical practice and from teaching other clinicians. Training included the rationale for the use of patient feedback and feedback scales, the practical use of the feedback scales, and how to incorporate the use of data from the scales in the treatment process.

The use of the PCOMS consisted of administering two feedback scales in every treatment session, one at the beginning of the session (the Outcome Rating Scale, or ORS), and one at the end (the Session Rating Scale, or SRS) [30]. Completing and scoring each scale takes less than one minute. Both scales consist of four questions scored on a 10 cm scale. In the ORS the patients rate their own functioning during the last week, or since the last treatment session, individually, interpersonally, socially, and generally. On the SRS, the patients rate the current session on relations with the therapist and the degree of agreement on goals, methods, and treatment approach.

Based on the patients’ initial ORS score, a progress curve was produced with a dotted line representing the expected trajectory of change for patients with similar ORS score in the first session [30]. The therapist had been trained to use this curve together with the patient to evaluate treatment progress. They had also learned to discuss the SRS scores with the patients to establish what was working in the session and what could be improved. The intervention thus consisted of systematically using the ORS and SRS scales to assess feedback from the patient on treatment outcome and the quality of the session.

To ensure fidelity in the PCOMS group, the patient feedback scales were collected after each consultation and the numbers of scales were compared to the total number of consultations registered on each participant in the clinic’s administrative data system. The controls received treatment as usual. To fortify fidelity in the treatment as usual group the therapists were repeatedly instructed to avoid using any feedback scales during treatment.

Both PCOMS and treatment as usual therapists were free to choose treatment approaches for their patients, and no recommendations or limitations were made, due to the study. The therapists thus chose the treatment approach they considered appropriate for each patient. The therapists working in the out-patient units in general mostly use psychotherapy, cognitive behavioural therapy, and some pharmacotherapy.

### Data collection/outcome measures

At baseline the participants completed a questionnaire on background information (Table 1). Outcome data were completed at baseline and six weeks after treatment started. Main outcome measures after six weeks of treatment were Treatment Alliance Scale (TAS) [40] and Client Satisfaction Questionnaire (CSQ) [44].

**Table 1:** Patients: total sample, intervention, and control

**Variables**	**Total sample**	**Intervention**	**Control**
	**N = 75**	**n = 37**	**n = 38**
	**N (%)**	**n (%)**	**n (%)**
*Female*	47 (62.7%)	26 (70.3%)	21 (55.3%)
*Age* (Mean (Median, Range))	29.9 (25, 18–70)	30.5 (26, 18–64)	29.2 (25, 20–70)
*Living alone*	23 (30.7%)	9 (24.3%)	14 (36.8%)
*Can confide in two or more persons*	54 (72.0%)	30 (81.1%)	24 (63.2%)
*Highest level of education*			
- Primary and lower secondary school	13 (17.3%)	9 (24.3%)	4 (10.5%)
- Upper secondary school	32 (42.7%)	12 (32.4%)	20 (52.6%)
- University	30 (40.0%)	16 (43.2%)	14 (36.8%)
*Working*	17 (22.7%)	11 (29.7%)	6 (15.8%)
*In education*	24 (32.0%)	11 (29.7%)	13 (34.2%)
*Currently using medication for mental health problems*	32 (42.7%)	14 (37.8%)	18 (47.4%)
*Previous treatment for mental health problems*	59 (78.7%)	29 (78.4%)	30 (78.9%)
*Previously hospitalized for mental health problems*	14 (18.7%)	8 (21.6%)	6 (15.8%)

Numbers are N/n (%) unless otherwise stated.

The Treatment Alliance Scale (TAS) is a 10-item scale measuring patients’ experience of alliance with therapists [40], providing a total score from 0 to 6 (strongest alliance). TAS was translated to Norwegian for the present study and we replaced “treatment team” with “therapist” in all items to make it suitable for out-patients. TAS has solid psychometric properties [40]. The Treatment Alliance Scale was chosen since it includes questions considered highly relevant; whether the patient experienced the therapist as respectful, understanding, attentive, helpful, and accessible. The questions also included the patients’ rating of collaboration, shared understanding, active participation, and whether the treatment would be helpful.

The Client Satisfaction Questionnaire-8 (CSQ-8) measures patient satisfaction [44]. The questionnaire includes eight items, providing a total score from 8 to 32 (highest satisfaction). The CSQ-8 has good psychometric properties [44,45], and was translated to Norwegian for the present study.

Several outcomes were added to investigate various aspects:

Since the use of the PCOMS are supposed to improve treatment results in mental health care, the Behaviour and Symptom Identification Scale 32 (BASIS-32) [46] was included. BASIS-32 is a 32 item questionnaire measuring mental health symptoms and functioning and provides a total score from 0 to 4 (worst mental health symptoms) [46]. BASIS-32 was translated to Norwegian for the present study.

Patient activation is a central aspect of an active patient role where patients and providers are more equal partners [47]. Patient Activation Measure (PAM) was therefore used to measure patient activation [47], providing a total PAM score ranging from 0 to 100 (highest activation). The validated Norwegian version was used [48].

Improved treatment results and patient activation would also imply improved health-related quality of life in patients, and Short Form-12v2 (SF-12v2) was therefore used to measure the patient’s self-reported health-related quality of life [49]. SF-12v2 is a recognised and much used health-related quality of life measurement. The 12 item scores are calculated into two total scores: The Mental Component Score (MCS), reflecting mental health-related quality of life, and the Physical Component Score (PCS), reflecting physical health-related quality of life. The total scores range from 0 to 100 (best quality of life). We used the Norwegian version of SF12v2 (SF-12v2™ Health Survey, 2004, Health Assessment Lab, Medical Outcomes Trust and Quality Metric Inc.).

Since all previous studies [34-36] investigating the effect of PCOMS have used the Outcome Rating Scale (ORS) as primary outcome, we included the ORS and the Session Rating Scale (SRS) as secondary outcome measures [30]. The total scores for each scale range from 0 to 40 (best). The Norwegian versions were used.

To measure user participation, 14 statements about participation and motivation, developed by the authors, were added. The statements were based on the literature and our own work on how patients and health personnel define patient participation [13]. Baseline data were used to conduct an exploratory factor analysis. Using varimax rotation, two factors were found to have an eigenvalue above 1.0, explaining nearly 63% of the variance. Three statements on motivation for treatment constituted one factor – “Patient motivation” (PM). Examples of statements on motivation are “I want to make a large effort to improve,” and “It is very important for me that my mental health problems improve”. The remaining 11 statements constituted the second factor – “Patient participation” (PP). Examples of statements on participation are “I am treated with respect from my therapist and others I have contact with during my treatment,” and “My therapist is interested in hearing my opinion.” Each statement was scored from 0 (strongly disagree) to 6 (strongly agree), and the total score was the mean of all statements (0 to 6, where 6 is the strongest motivation and most participation).

For the translations of TAS, CSQ, and BASIS-32, two persons independently translated the questions from English to Norwegian, and two other persons independently translated them from Norwegian to English. Lack of accordance was discussed until consensus was reached. The Norwegian versions were tested on volunteers. Effort was made to keep the Norwegian versions similar to the originals.

### Power calculation and sample size

Due to the lack of similar studies on treatment alliance, the sample size was determined using the assumed clinically significant difference in the BASIS-32 questionnaire (mental health symptoms and functioning). To detect a clinically significant difference between the groups’ BASIS-32 scores of 0.5 with a standard deviation of 0.8 [50], 32 participants were needed in each group (power 0.8, alpha 0.05). No interim analyses were planned or conducted.

### Randomisation and allocation

After completing baseline data, participants were randomised to either PCOMS or treatment as usual, using the university’s internet based computerised randomisation service. There was no stratification or block randomisation. Patients were subsequently allocated to a PCOMS therapist or treatment as usual therapist by the unit manager. Therapists could thus not influence which patients they treated.

### Blinding

This was an open study and no blinding was performed.

### Statistical methods

For all questionnaires, only those with at least 50% of the items answered valid were scored. Missing values were replaced with last value (baseline) carried forward, or if no baseline values were available, missing values were replaced with the mean value of the group the patient was randomised to. Information on the number of consultations for three patients in the intervention group and nine in the control group was not accessible from the clinic’s administrative data system. These missing data were not replaced. Analyses both with and without missing data were conducted and the results were the same.

Both intention to treat and per protocol analyses were conducted [51]. The pre-defined criterion for per protocol analysis was that patients should have attended at least three consultations during six weeks from the initial consultation. This would ensure that all patients received a minimum of treatment. In addition, the PCOMS group should have used the feedback scales in at least 2/3 of all consultations to ensure that the PCOMS group received the intervention.

Between group differences for TAS, CSQ, SRS, and PP were analysed using two-tailed independent t-tests. Between group differences for the other outcomes were analysed using analysis of covariance (ANCOVA) with the baseline value as a covariate. Within group differences (from baseline to six weeks) were calculated using two-tailed paired t-tests. A significance level of 5% (p ≤ 0.05) was chosen to calculate confidence intervals (95% CI). Analyses were done with SPSS 17.0 for Windows (SPSS Inc., Chicago, IL).

## Results

### Participant flow

**Figure 1:**
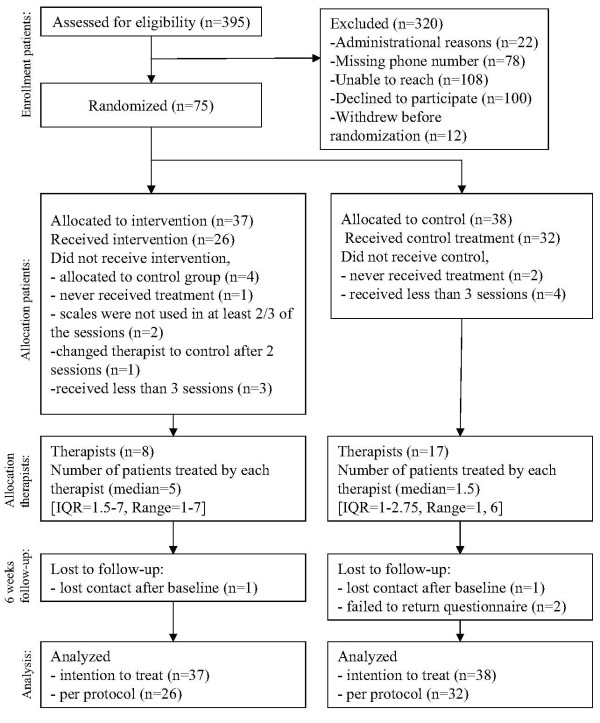
Flow chart study.

The flow of participants during recruitment and study is described in Figure 1. Three hundred and ninety-five out-patients were eligible and received an invitation letter. Eighty-seven volunteered to participate and 12 withdrew before randomisation. A total of 75 patients were randomised; 37 were put in the PCOMS group and 38 in the treatment as usual group.

### Baseline data

#### Patients

In the total sample 63% were female and the mean age was 30 years (Table 1). 31% lived alone, and 72% had two or more persons they could confide in. 79% had previous treatment experience for mental health problems, and 43% were currently using medication for such problems.

Age, gender, and referral diagnoses were collected consecutively for 40% (n = 157) of the total group of patients who were eligible and invited to, but did not participate. Due to data registration restrictions, we were not able to collect data for all non-participating patients. The mean age was 28.4 (range 18–71), and 58% were female. Diagnoses at referral were also similar with the patients who participated, with anxiety and depression as the main diagnosis (data not shown).

#### Therapists

Eight therapists treated the patients in the PCOMS group, while 17 treated the patients in the treatment as usual group (Table 2). Four of the 17 therapists in the treatment as usual group did not respond to a questionnaire about therapists’ characteristics, and these missing values were not replaced. 64% of the therapists were female, 76% were psychologists and 24% were psychiatric nurses. The mean age was 41 years, they had 1–16 years of experience with out-patient mental health treatment, and 1–25 years of experience from mental health care in general. 43% had heard about the PCOMS before, and 9.5% had previously attended a course in using the PCOMS in clinical work. The number of therapists participating was 86% of the total number of therapists in the outpatient unit at the hospital who provided individual treatment. The two groups of therapist were similar regarding gender, education, age, and work experience. The PCOMS therapists treated a mean of 4.4 patients each (median 5, range 1–7), while the treatment as usual therapists treated a mean of 2.4 patients each (median 2, range 1–6).

**Table 2:** Therapists; total sample, intervention, and control

**Variables**	**Total sample**	**Intervention**	**Control**	**p-value**
	**N = 25**	**n = 8**	**n = 17**	
	**N (%)**	**n (%)**	**n (%)**	
*Female*	16 (64%)	5 (62.5%)	11 (64.7%)	0.915
*Age* (Mean (Median, Range))	41.4 (38, 30–62)	39 (34.5, 31–62)	42.9 (41, 30–61)	0.402
*Education*				0.920
- Psychiatric nurse	5 (23.8%)	2 (25%)	3 (17.6%)	
- Psychologist	16 (76.2%)	6 (75%)	10 (58.8%)	
*No of years worked in mental health care* (Mean (Median, Range))	9.9 (9, 1–25)	7.4 (5.5, 1–16)	11.4 (10, 1–25)	0.220
*No of years worked in out-patient unit* (Mean (Median, Range))	4.6 (3, 1–16)	3.4 (3, 1–8)	5.4 (4, 1–16)	0.292
*Heard about PCOMS prior to study* (yes)	9 (42.9%)	3 (37.5%)	6 (35.3%)	0.697
*Attended course in PCOMS prior to study* (yes)	2 (9.5%)	1 (12.5%)	1 (7.7%)	0.716

Numbers are N/n (%) unless otherwise stated.

For categorical variables the groups have been compared using Pearson’s chi square tests.

For continuous variables the groups have been compared using two-tailed independent t-tests.

Four treatment as usual therapists did not return the questionnaire on these variables.

### Implementation of intervention

During the six weeks, the patients in the PCOMS group had an average of 3.8 (range 1–9) consultations. The patients in the treatment as usual group had an average of 3.8 (range 1–10) consultations. In the PCOMS group the scales were used in 92% of all consultations.

### Comparison between the groups

The differences between the PCOMS group and treatment as usual group on all outcome measures are presented in Table 3.

**Table 3:** Intention to treat analyses

**Outcomes**	**Group**	**Baseline**	**Between groups at 6 weeks A)**			**Within groups (6 weeks–baseline) B)**	
		**Mean (SD)**	**Mean (SD)**	**Diff. (95% CI)**	**p-value**	**Diff (95% CI)**	**p-value**
**TAS**	INT		4.63 (1.1)	0.08 (−0.44, 0.59)	0.760		
	CTRL		4.55 (1.1)				
**CSQ**	INT		24.42 (5.0)	0.24 (−1.85, 2.32)	0.819		
	CTRL		24.18 (4.0)				
**BASIS-32**^**†**^	INT	1.05 (0.5)	1.00 (0.5)	0.02 (−0.20, 0.24)	0.882	−0.05 (−0.21, 0.12)	0.551
	CTRL	1.23 (0.5)	1.08 (0.6)			−0.15, (−0.33, 0.03)	0.107
**PAM**	INT	35.36 (9.1)	38.58 (7.7)	1.58 (−2.28, 5.44)	0.417	3.22 (0.70, 5.74)*	0.014
	CTRL	36.09 (9.1)	37.34 (10.8)			1.25 (−2.43, 4.94)	0.495
**MCS (SF-12)**	INT	38.19 (8.4)	40.63 (9.2)	0.03 (−3.5, 3.6)	0.989	2.44 (−0.49, 5.38)	0.100
	CTRL	38.08 (5.9)	40.55 (7.9)			2.47 (−0.21, 5.16)	0.070
**PCS (SF-12)**	INT	48.05 (7.6)	49.10 (8.9)	0.92 (−2.05, 3.89)	0.539	1.04 (−1.06, 3.14)	0.321
	CTRL	48.29 (7.9)	48.35 (8.7)			0.69 (−2.22, 2.36)	0.952
**ORS**	INT	18.64 (8.2)	22.17 (9.2)	1.73 (−1.89, 5.35)	0.344	3.53 (0.99, 6.06)*	0.008
	CTRL	16.83 (6.7)	19.33 (8.8)			2.51 (−0.41, 5.43)	0.090
**SRS**	INT		31.88 (7.9)	1.46 (−2.28, 5.19)	0.440		
	CTRL		30.43 (8.3)				
**PM**	INT	5.34 (0.8)	5.59 (0.6)	0.29 (0.00, 0.57)*	0.050	0.25 (−0.06, 0.57)	0.113
	CTRL	5.31 (0.8)	5.30 (0.8)			−0.02 (−0.23, 0.19)	0.874
**PP**	INT		4.43 (1.0)	−0.09 (−0.52, 0.34)	0.663		
	CTRL		4.52 (0.9)				

Comparison of outcomes between the intervention group (INT, n = 37) and control group (CTRL, n = 38) six weeks after treatment start, and within each group from baseline to six weeks.

TAS = Treatment alliance score, CSQ = Client satisfaction questionnaire, BASIS-32 = The behavior and symptom identification scale 32, PAM = Patient activation measure, MCS (SF-12) = Mental Component Score (Short form-12), PCS (SF-12) = Physical Component Score (Short form-12), ORS = Outcome rating scale, SRS = Session rating scale, PM = Patient motivation, PP = Patient participation.

A) The between-group mean differences for TAS, CSQ, SRS and PP have been calculated using two-tailed independent t-tests. The between-group estimated mean differences for BASIS-32, PAM, SF12, ORS, and PM have been calculated using analysis of covariance (ANCOVA), adjusted for baseline values.

B) The within group differences have been calculated using two-tailed paired *t*-test.

^**†**^ Decrease in score indicates improvement (less mental health symptoms) for BASIS-32. For the other outcomes, increased score indicates improvement.

* p-value ≤0.05.

There were no differences between the groups on treatment alliance (TAS) (mean difference = 0.08, 95% CI −0.44 to 0.59, p = 0.760) and patient satisfaction (CSQ) (mean difference = 0.24, 95% CI −1.85 to 2.32, p = 0.819) six weeks after the treatment started.

There was a higher score on motivation for treatment (PM) in the PCOMS group (estimated mean difference = 0.29, 95% CI 0.00 to 0.57, p = 0.05). There were no differences on the other outcomes.

### Changes within the groups

The changes in means from baseline to six weeks after starting treatment, for both the PCOMS group and treatment as usual group, are presented in Table 3 (last 2 columns). Since it was not possible to complete the outcomes on alliance (TAS), satisfaction (CSQ), treatment sessions (SRS), and patient participation (PP) before starting treatment, no changes within groups could be calculated for these variables. Two outcomes had improvement in the PCOMS group; patient activation (PAM, mean diff = 3.22, 95% CI 0.70 to 5.74, p = 0.014) and patient functioning (ORS, mean diff = 3.53, 95% CI 0.99 to 6.06, p = 0.008). There were no differences in the treatment as usual group.

### Per protocol analyses

Per protocol analyses were conducted only on participants who received the intervention they were randomised to according to protocol. The predefined per protocol criteria gave 26 participants in the intervention group and 32 in the control group. Reasons for not meeting the criteria in the PCOMS group were being mistakenly allocated to the control group (four patients), never starting treatment (one patient), not using scales in at least 2/3 of the treatment sessions (two patients), changing therapist to control after two sessions (one patient), and receiving less than three sessions (three patients). Reasons for not meeting the criteria in the treatment as usual group were never starting treatment (two patients) and receiving less than three treatment sessions (four patients). Patients meeting per protocol criteria received an average of 4.0 treatment sessions during six weeks.

The per protocol analyses showed no differences between the PCOMS group and the treatment as usual group on alliance or satisfaction, six weeks after starting treatment (Table 4), but, compared to the intention to treat analysis, the absolute values tended to change in favour of the PCOMS group. The effect size [52] increased from 0.07 (intention to treat, Table 3) to 0.40 (per protocol, Table 4) for treatment alliance and from 0.06 to 0.31 for patient satisfaction.

**Table 4:** Per protocol analyses

**Outcomes**	**Group**	**Baseline**	**Between groups at 6 weeks A)**			**Within groups (6 weeks–baseline) B)**	
		**Mean (SD)**	**Mean (SD)**	**Diff. (95% CI)**		**Diff (95% CI)**	**p-value**
**TAS**	INT		5.01 (0.8)	0.32 (−0.11, 0.75)	0.137		
	CTRL		4.69 (0.8)				
**CSQ**	INT		25.59 (4.0)	1.16 (−0.84, 3.16)	0.248		
	CTRL		24.43 (3.6)				
**BASIS-32**^**†**^	INT	1.10 (0.5)	1.03 (0.5)	0.04 (−0.22, 0.30)		−0.07 (−0.27, 0.14)	0.515
	CTRL	1.27 (0.6)	1.09 (0.6)		0.766	−0.18 (−0.37, 0.01)	0.069
**PAM**	INT	35.17 (6.4)	39.43 (7.0)	2.53 (−2.04, 7.10)	0.272	4.26 (1.71, 6.81)*	0.002
	CTRL	36.17 (8.8)	37.29 (10.5)			1.12 (−3.15, 5.40)	0.596
**MCS (SF-12)**	INT	37.41 (7.5)	39.94 (7.8)	−0.29 (−4.21, 3.63)	0.883	2.54 (−0.40, 5.48)	0.088
	CTRL	37.37 (6.1)	40.21 (8.5)			2.84 (−0.23, 5.92)	0.069
**PCS (SF-12)**	INT	47.46 (8.4)	48.84 (9.9)	1.42 (−2.20, 5.04)	0.434	1.38 (−1.48, 4.24)	0.330
	CTRL	48.09 (8.6)	47.90 (9.0)			−0.18 (−2.70, 2.34)	0.883
**ORS**	INT	17.55 (7.0)	22.07 (9.0)	2.68 (−1.74, 7.09)	0.229	4.52 (1.31, 7.73)*	0.008
	CTRL	16.16 (7.0)	18.63 (9.2)			2.47 (−0.94, 5.89)	0.150
**SRS**	INT		34.68 (5.6)	3.15 (0.07, 6.37)	0.055		
	CTRL		31.53 (6.4)				
**PM**	INT	5.38 (0.8)	5.65 (0.5)	0.28 (0.04, 0.42)	0.024	0.27 (−0.04, 0.57)	0.081
	CTRL	5.32 (0.9)	5.34 (0.7)			0.02 (−0.14, 0.19)	0.788
**PP**	INT		4.73 (0.7)	0.07 (−0.29, 0.42)	0.706		
	CTRL		4.66 (0.6)				

Comparison of outcomes between the intervention group (INT, n = 26) and control group (CTRL, n = 32) six weeks after treatment start, and within each group from baseline to six weeks.

TAS = Treatment alliance score, CSQ = Client satisfaction questionnaire, BASIS-32 = The behavior and symptom identification scale 32, PAM = Patient activation measure, MCS (SF-12) = Mental Component Score (Short form-12), PCS (SF-12) = Physical Component Score (Short form-12), ORS = Outcome rating scale, SRS = Session rating scale, PM = Patient motivation, PP = Patient participation.

A) The between-group mean differences for TAS, CSQ, SRS and PP have been calculated using two-tailed independent t-tests. The between-group estimated mean differences for BASIS-32, PAM, SF12, ORS, and PM have been calculated using analysis of covariance (ANCOVA), adjusted for baseline values.

B) The within group differences have been calculated using two-tailed paired *t*-test.

^**†**^ Decrease in score indicates improvement (less mental health symptoms) for BASIS-32. For the other outcomes, increased score indicates improvement.

* p-value ≤0.05.

The PCOMS group had higher scores than the treatment as usual group on motivation for treatment (PM) (estimated mean diff = 0.28, 95% CI 0.04 to 0.52, p = 0.024), and near significant higher scores on evaluation of consultations (SRS) in the PCOMS group (mean diff = 3.15, 95% CI 0.07 to 6.37, p = 0.055).

For within group analysis, PAM and ORS improved significantly in the PCOMS group, as in the intention to treat analysis.

## Discussion

### Results

Six weeks after starting treatment, no significant effects on treatment alliance and patient satisfaction from using the PCOMS scales were found. Both hypotheses were thus rejected. The PCOMS group had higher motivation for treatment compared with the treatment as usual group, and there were improvements within the PCOMS group of patient activation (PAM) and patient functioning (ORS).

### Strengths and limitations

The main strength of this study is that it is the first randomised controlled trial on the effect on treatment alliance and patient satisfaction of systematically using the two PCOMS scales in treatment sessions. The study was conducted in a natural setting. The sample of patients participating in this study was similar in gender, age, and referral diagnoses to a larger sample from this hospital out-patient unit, indicating that the study sample were representative of those referred to out-patient mental health treatment. Although the therapists initially self-selected to participate, 86% of all eligible therapists at the out-patient units eventually participated in the study. The results therefore have strong external validity. The mean ORS score at baseline for the participants in the study was 18.8. Since this score is well below 25, which is considered the cut-off for those in need of treatment [30], this was a suitable sample from mental health care.

The high adherence to the use of the scales, which was used in 92% of the consultations, confirms that the intervention was delivered as planned. To fortify fidelity to the study design, the treatment as usual therapists were repeatedly instructed to avoid using any feedback scales in the consultations, but the consultations were not video or audio taped. We found no indications that feedback scales were used in the treatment as usual group.

The training the PCOMS therapists received was similar to previous studies [34-37], and in accordance with training given for clinical purposes to improve patient functioning and progress [43]. According to Miller and colleagues [30], patients using PCOMS should have a change of at least 5 points on the ORS score to show reliable change after three consultations. In the present study, the mean change after three consultations was 6.6 points, indicating that the sample followed the expected treatment progress.

The size of the study might be a limitation. Given the results for treatment alliance in the per protocol analysis (difference in TAS = 0.32, standard deviation 0.8) and that additional patients had got the same results, a total of 113 patients in each group would be needed to get a result with a p-value < 0.05 (power 0.8, alpha 0.05).

Cronbach’s alpha was calculated and found satisfactory for the outcome measures that were translated to Norwegian for this study. The results were 0.89 for BASIS-32 (mental health symptoms and functioning), 0.93 for TAS (treatment alliance), and 0.96 for CSQ (patient satisfaction).

### Alliance and satisfaction

We hypothesised that, six weeks after starting treatment, treatment alliance and patient satisfaction would be higher in the PCOMS group than the treatment as usual group. Both hypotheses were rejected. That there were no effects on alliance or satisfaction in the present study questions if the alleged increase in alliance when using the PCOMS scales is due to treatment in general or feedback techniques embedded in treatment, and not the use of the scales. The observation that the differences between the groups in the per protocol analysis (where only those who used the scales as intended were included) was more in favour of the PCOMS group than in the intention to treat analysis, warrants a larger study to draw a more firm conclusion.

The instrument used to measure treatment alliance in this study (Treatment Alliance Scale (TAS)) has been relatively little used and tested so far, but was chosen since it is a validated questionnaire which is easy to complete. More used instruments like The Revised Helping Alliance Questionnaire [53] and The Working Alliance Inventory [54] could have been chosen. However, a meta-analysis on alliance questionnaires reported no difference in ability to measure alliance across instruments [24].

Since the Session Rating Scale (SRS) is the patient’s rating of the relation with the therapist and the degree of agreement on goals, methods, and treatment approach, it has similarities to a treatment alliance rating. In the present study, the per protocol analysis showed that the PCOMS group had a higher score on SRS that was near to a p-value of 0.05. This could indicate that the feedback scales have an effect on an alliance-related dimension, which is encompassed by the SRS but not the TAS. The results on the SRS have to be interpreted with caution since the PCOMS group was exposed to the scales in the treatment sessions, and this might have influenced their answers.

Since the most important factor affecting patient satisfaction is the relationship between patient and professional [16], it is reasonable that the results on treatment alliance and patient satisfaction in the present study were consistent. The lack of effect on patient satisfaction is in line with previous investigations where clinicians have been provided patient ratings of health status or quality of life [17,18].

### Motivation

The present study found that the PCOMS group had higher motivation for treatment than the treatment as usual group, six weeks after starting treatment. Patients’ expectations of treatment effect have been shown by some to be important predictors of treatment outcome [55], while others have shown that motivation does not predict treatment result [56]. It has been argued that motivation is an internal patient factor which cannot be influenced, while others highlight social factors as important for changing motivation [57]. Research has shown that motivation correlates with alliance at treatment start [58]. In the literature, motivation has been linked to setting and revising goals, encouraging the patient’s view, and welcoming the patient’s value system [57]. These factors are central when using patient feedback scales.

### Treatment outcome

Previous randomised controlled trials have shown that use of PCOMS was superior to treatment as usual after between 5 and 8 sessions [34-36]. These studies have used the ORS scale as the main outcome measure. However, in the present study there were no short-term differences between the PCOMS group and the treatment as usual group regarding the ORS scores. This could be due to the shorter follow up time in this study. Nevertheless, the mean ORS score for the PCOMS group followed the predicted score during the consultations, indicating that this group had good progress during treatment [30]. Furthermore, there was a clear change in the ORS scores from baseline to six week follow-up within the PCOMS group (mean difference = 3.53, 95% CI 0.99 to 6.06, p = 0.008), and not within the treatment as usual group (mean difference = 2.51, -0.41 to 5.43, p = 0.090). A longer follow up time is needed to make more exact comparisons with other studies investigating the effect of use of PCOMS.

## Conclusion

The present study is the first randomised controlled trial investigating the short-term effect on treatment alliance and patient satisfaction of using the PCOMS scales in out-patient treatment. Compared to treatment as usual, there were no effects on alliance and satisfaction from using the scales six weeks after starting treatment. The PCOMS group had higher motivation for treatment. In addition, the per protocol analyses showed higher effect sizes. Future investigations in a larger study are therefore warranted.

## Competing interests

The authors declare that they have no competing interests. HG is a member of the board of the hospital trust where the study took place.

## Authors’ contributions

MBR designed and implemented the study, collected, analyzed and interpreted data, and wrote and completed the manuscript. LE contributed to the design and implementation of the study, to the interpretation of data, and to the writing and completion of the manuscript. HG contributed to the interpretation of data, and to the writing and completion of the manuscript. AS designed and supervised the implementation of the study, interpreted data, and contributed to the writing and completion of the manuscript. All authors read and approved the final manuscript.

## Pre-publication history

The pre-publication history for this paper can be accessed here:

http://www.biomedcentral.com/1472-6963/12/348/prepub

## References

[B1] Longtin Y Sax H Leape LL Sheridan SE Donaldson L Pittet D Patient participation: current knowledge and applicability to patient safety Mayo Clin Proc 2010 85 53 62 10.4065/mcp.2009.0248 20042562 PMC2800278

[B2] May C Montori VM Mair FS We need minimally disruptive medicine BMJ 2009 339 b2803 10.1136/bmj.b2803 19671932

[B3] Crawford M Rutter D Manley C Weaver T Bhui K Fulop N Systematic review of involving patients in the planning and development of health care BMJ 2002 325 1263 10.1136/bmj.325.7375.1263 12458240 PMC136920

[B4] Coulter A Ellins J Effectiveness of strategies for informing, educating, and involving patients BMJ 2007 335 24 27 10.1136/bmj.39246.581169.80 17615222 PMC1910640

[B5] Cegala DJ Street RL Jr Clinch CR The impact of patient participation on physicians' information provision during a primary care medical interview Health Commun 2007 21 177 185 10.1080/10410230701307824 17523863

[B6] Tritter JQ Revolution or evolution: the challenges of conceptualizing patient and public involvement in a consumerist world Health Expect 2009 12 275 287 10.1111/j.1369-7625.2009.00564.x 19754691 PMC5060496

[B7] Florin D Dixon J Public involvement health BMJ 2004 328 159 161 10.1136/bmj.328.7432.159 14726350 PMC314519

[B8] Carr S Participation, power, conflict and change: theorizing dynamics of service user participation in the social care system of England and Wales CSP 2007 27 266 276

[B9] Stringer B Van Meijel B De Vree W Van der Bijl J User involvement in mental health care: the role of nurses. a literature review J Psychiatr Ment Health Nurs 2008 15 678 683 10.1111/j.1365-2850.2008.01285.x 18803743

[B10] Hickey G Kipping C Exploring the concept of user involvement in mental health through a participation continuum J Clin Nurs 1998 7 83 88 10.1046/j.1365-2702.1998.00122.x 9510712

[B11] Coulter A The autonomous patient 2002 London: Nuffield Trust

[B12] Florin J Ehrenberg A Ehnfors M Patient participation in clinical decision-making in nursing: a comparative study of nurses' and patients' perceptions J Clin Nurs 2006 15 1498 1508 10.1111/j.1365-2702.2005.01464.x 17118072

[B13] Rise MB Solbjor M Lara MC Westerlund H Grimstad H Steinsbekk A Same description, different values. how service users and providers define patient and public involvement in health care Health Expect 2011 [Epub ahead of print] 10.1111/j.1369-7625.2011.00713.x PMC5060665 21838833

[B14] Cahill J Patient participation-a review of the literature J Clin Nurs 1998 7 119 128 10.1111/j.1365-2702.1998.00132.x 9582762

[B15] Happell B Pinikahana J Roper C Attitudes of postgraduate nursing students towards consumer participation in mental health services and the role of the consumer academic Int J Ment Health Nurs 2002 11 240 250 10.1046/j.1440-0979.2002.00255.x 12664455

[B16] Crow R Gage H Hampson S Hart J Kimber A Storey L The measurement of satisfaction with healthcare: implications for practice from a systematic review of the literature Health Technol Assess 2002 6 1 244 12925269 10.3310/hta6320

[B17] Haywood K Marshall S Fitzpatrick R Patient participation in the consultation process: a structured review of intervention strategies Patient Educ Couns 2006 63 12 23 10.1016/j.pec.2005.10.005 16406464

[B18] Marshall S Haywood K Fitzpatrick R Impact of patient-reported outcome measures on routine practice: a structured review J Eval Clin Pract 2006 12 559 568 10.1111/j.1365-2753.2006.00650.x 16987118

[B19] Bjertnaes OA Sjetne IS Iversen HH Overall patient satisfaction with hospitals: effects of patient-reported experiences and fulfilment of expectations BMJ Qual Saf 2011 10.1136/bmjqs-2011-000137 21873465

[B20] Svensson B Hansson L Satisfaction with mental health services. a user participation approach Nord J Psychiatry 2006 60 365 371 10.1080/08039480600937090 17050294

[B21] Gelso C Carter J Components of the psychotherapy relationship J Couns Psychol 1994 41 296 306

[B22] Gelso C Carter J The relationship in counseling and psychotherapy: Components, consequences, and theoretical antecedents TCP 1985 13 155 243

[B23] Bordin E The generalizability of the psychoanalytic concept of the working alliance Psychotherapy (Chic) 1979 16 252 260

[B24] Martin DJ Garske JP Davis MK Relation of the therapeutic alliance with outcome and other variables: a meta-analytic review J Consult Clin Psychol 2000 68 438 450 10883561

[B25] Howard KI Moras K Brill PL Martinovich Z Lutz W Evaluation of psychotherapy. efficacy, effectiveness, and patient progress Am Psychol 1996 51 1059 1064 8870542 10.1037//0003-066x.51.10.1059

[B26] Miller SD Duncan BL Brown J Sorrell R Chalk MB Using formal client feedback to improve retention and outcome: making ongoing, real-time assessment feasible J Brief Therapy 2006 5 5 22

[B27] Harmon SC Lambert MJ Smart DM Hawkins E Nielsen SL Slade K Enhancing outcome for potential treatment failures: therapist client feedback and clinical support tools Psychother Res 2007 17 379 392 10.1080/10503300600702331

[B28] Lambert MJ Harmon C Slade K Whipple JL Hawkins EJ Providing feedback to psychotherapists on their patients' progress: clinical results and practice suggestions J Clin Psychol 2005 61 165 174 10.1002/jclp.20113 15609358

[B29] Lambert MJ Shimokawa K Collecting client feedback Psychotherapy (Chic) 2011 48 72 79 21401277 10.1037/a0022238

[B30] Miller SD Duncan BL Sorrell R Brown GS The partners for change outcome management system J Clin Psychol 2005 61 199 208 10.1002/jclp.20111 15609362

[B31] Tritter JQ McCallum A The snakes and ladders of user involvement: moving beyond Arnstein Health Policy 2006 76 156 168 10.1016/j.healthpol.2005.05.008 16006004

[B32] Arnstein S A ladder of citizen participation J Am Plann Assoc 1969 35 216 224 10.1080/01944366908977225

[B33] Lambert M Whipple J Harmon C Shimokawa K Slade K Christofferson C Clinical support tools manual 2004 Brigham Young University, Provo UT: Department of Psychology

[B34] Reese R Norsworthy L Rowlands S Does a continuous feedback system improve psychotherapy outcome? Psychotherapy (Chic) 2009 46 418 431 22121836 10.1037/a0017901

[B35] Reese RJ Toland MD Slone NC Norsworthy LA Effect of client feedback on couple psychotherapy outcomes Psychotherapy (Chic) 2010 47 616 630 21198247 10.1037/a0021182

[B36] Anker M Duncan B Sparks J Using client feedback to improve couple therapy outcomes: a randomized clinical trial in a naturalistic setting J Consult Clin Psychol 2009 77 693 704 19634962 10.1037/a0016062

[B37] Reese R Usher E Bowman D Norsworthy L Halstead J Rowlands S Using client feedback in psychotherapy training: an analysis of its influence on supervision and counselor self-efficacy TEPP 2009 3 157 168

[B38] Horvath AO Del Re AC Fluckiger C Symonds D Alliance in individual psychotherapy Psychotherapy (Chic) 2011 48 9 16 21401269 10.1037/a0022186

[B39] Johansson H Eklund M Helping alliance and early dropout from psychiatric out-patient care: the influence of patient factors Soc Psychiatry Psychiatr Epidemiol 2006 41 140 147 10.1007/s00127-005-0009-z 16372143

[B40] Blais MA Development of an inpatient treatment alliance scale J Nerv Ment Dis 2004 192 487 493 10.1097/01.nmd.0000131911.53489.af 15232319

[B41] Beach M Sugarman J Johnson RL Arbelaez JJ Duggan PS Cooper LA Do patients treated with dignity report higher satisfaction, adherence, and receipt of preventive care? Ann Fam Med 2005 3 331 338 10.1370/afm.328 16046566 PMC1466898

[B42] Joffe S Manocchia M Weeks JC Cleary PD What do patients value in their hospital care? an empirical perspective on autonomy centred bioethics J Med Ethics 2003 29 103 108 10.1136/jme.29.2.103 12672891 PMC1733711

[B43] Miller SD Duncan BL The outcome and session rating scales: administration and scoring manual 2004 Chicago, IL: Scott Miller & Barry Duncan

[B44] Larsen DL Attkisson CC Hargreaves WA Nguyen TD Assessment of client/patient satisfaction: development of a general scale Eval Program Plann 1979 2 197 207 10.1016/0149-7189(79)90094-6 10245370

[B45] Attkisson C Zwick R The client satisfaction questionnaire. Psychometric properties and correlations with service utilization and psychotherapy outcome Eval Program Plann 1982 5 233 237 10.1016/0149-7189(82)90074-X 10259963

[B46] Eisen SV Dill DL Grob MC Reliability and validity of a brief patient-report instrument for psychiatric outcome evaluation Hosp Commun Psych 1994 45 242 247 10.1176/ps.45.3.242 8188195

[B47] Hibbard JH Stockard J Mahoney ER Tusler M Development of the Patient Activation Measure (PAM): conceptualizing and measuring activation in patients and consumers Health Serv Res 2004 39 1005 1026 10.1111/j.1475-6773.2004.00269.x 15230939 PMC1361049

[B48] Steinsbekk A [Patient Activation Measure] Tidsskr Nor Laegeforen 2008 128 2316 2318 19096487

[B49] Ware J Jr Kosinski M Keller SD A 12-Item Short-Form Health Survey: construction of scales and preliminary tests of reliability and validity Med Care 1996 34 220 233 10.1097/00005650-199603000-00003 8628042

[B50] Clements K Murphy J Eisen S Normand S Comparison of self-report and clinician-rated measures of psychiatric symptoms and functioning in predicting 1-year hospital readmission Adm Policy Ment Health 2006 33 568 577 10.1007/s10488-006-0066-y 16799832

[B51] Altman D Bland J Interaction revisited: the difference between two estimates BMJ 2003 326 219 10.1136/bmj.326.7382.219 12543843 PMC1125071

[B52] Cohen J A Power Primer Psychol Bull 1992 112 155 159 19565683 10.1037//0033-2909.112.1.155

[B53] Luborsky L Barber JP Siqueland L Johnson S Najavits LM Frank LM The revised helping alliance questionnaire (HAq-II): psychometric properties J Psychother Pract Res 1996 5 260 271 22700294 PMC3330423

[B54] Horvath AO Greenberg LS Development and validation of the working alliance inventory J Couns Psychol 1989 36 223 233

[B55] Meyer B Pilkonis PA Krupnick JL Egan MK Simmens SJ Sotsky SM Treatment expectancies, patient alliance, and outcome: further analyses from the National Institute of Mental Health Treatment of Depression Collaborative Research Program J Consult Clin Psychol 2002 70 1051 1055 12182269

[B56] Vogel PA Hansen B Stiles TC Gotestam KG Treatment motivation, treatment expectancy, and helping alliance as predictors of outcome in cognitive behavioral treatment of OCD J Behav Ther Exp Psychiatry 2006 37 247 255 10.1016/j.jbtep.2005.12.001 16460667

[B57] Maclean N Pound P A critical review of the concept of patient motivation in the literature on physical rehabilitation Soc Sci Med 2000 50 495 506 10641802 10.1016/s0277-9536(99)00334-2

[B58] Johansson H Jansson JA Therapeutic alliance and outcome in routine psychiatric out-patient treatment: patient factors and outcome Psychotherapy (Chic) 2010 83 193 206 10.1348/147608309X472081 19793413

